# Colorectal cancer cells display chaperone dependency for the unconventional prefoldin URI1

**DOI:** 10.18632/oncotarget.8816

**Published:** 2016-04-18

**Authors:** Kamil Andrzej Lipinski, Christian Britschgi, Karen Schrader, Yann Christinat, Lukas Frischknecht, Wilhelm Krek

**Affiliations:** ^1^ Institute of Molecular Health Sciences, ETH Zurich, 8093 Zurich, Switzerland

**Keywords:** URI1, prefoldin, p53, chaperone dependency, colorectal cancer

## Abstract

Chaperone dependency of cancer cells is an emerging trait that relates to the need of transformed cells to cope with the various stresses associated with the malignant state. *URI1* (unconventional prefoldin RPB5 interactor 1) encodes a member of the prefoldin (PFD) family of molecular chaperones that acts as part of a heterohexameric PFD complex, the URI1 complex (URI1_C_), to promote assembly of multiprotein complexes involved in cell signaling and transcription processes. Here, we report that human colorectal cancer (CRCs) cell lines demonstrate differential dependency on URI1 and on the URI1 partner PFD STAP1 for survival, suggesting that this differential vulnerability of CRC cells is directly linked to URI1_C_ chaperone function. Interestingly, in URI1-dependent CRC cells, URI1 deficiency is associated with non-genotoxic p53 activation and p53-dependent apoptosis. URI1-independent CRC cells do not exhibit such effects even in the context of wildtype p53. Lastly, in tumor xenografts, the conditional depletion of URI1 in URI1-dependent CRC cells was, after tumor establishment, associated with severe inhibition of subsequent tumor growth and activation of p53 target genes. Thus, a subset of CRC cells has acquired a dependency on the URI1 chaperone system for survival, providing an example of ‘non-oncogene addiction’ and vulnerability for therapeutic targeting.

## INTRODUCTION

The progression of normal towards malignant tumor cells is invariably associated with increased proteotoxic stress perpetrated, in part, by the acquisition of genomic abnormalities (e.g. mutations, aneuploidy, copy-number variations) and the tumor microenvironment (e.g. hypoxia, acidosis). The consequent imbalances in protein expression, production of mutant proteins and assembly of multiprotein complexes with altered molecular composition and subunit stoichiometry creates a high demand for molecular chaperone systems to guard against protein misfolding, aggregation and promiscuous and dysfunctional protein-protein interactions [1]. Accordingly, tumor cells exhibit increased dependency on molecular chaperone systems for their survival, an observation particularly well illustrated in the case of the heat-shock protein (HSP) families HSP90 and HSP70 that are frequently overexpressed in human cancer and display marked anti-apoptotic activities and potent tumor-promoting activities [2].

Prefoldins (PFDs) are members of an evolutionary conserved protein family of molecular chaperones with roles in *de novo* protein folding, inhibition of protein aggregate formation and assembly of multiprotein complexes involved in cell signaling and transcription processes [3]. The human genome encodes nine PFDs with N- and C-terminal α-helical coiled-coil structures connected by either one (β-class PFDs) or two (α-class PFDs) β-hairpins that can assemble with an α2β4 subunit stoichiometry into hetero-hexameric complexes [4]. Two major hexameric PFD complexes have been described to date in mammalian cells and include the ‘prefoldin/GimC’ complex and the prefoldin-like ‘unconventional prefoldin RPB5-interactor (URI)1′ complex (URI1_C_) [5–7]. Prefoldin/GimC is composed of PFDs 1 to 6. URI1_C_ encompasses the α-class PFDs URI1 and STAP1 (SKP2-associated alpha PFD [5]; also referred to as UXT [8] or ART-27 [9]) and the β-class PFDs PFD2, PFD6 and PFD4-related (PFD4r [5]; also referred to as p53 and DNA damage-regulated 1 [PDRG1] [10]). It is assumed that in the URI1_C_ one member of the β-class PFDs is present in two copies to satisfy a α2β4 subunit stoichiometry. Both prefoldin/GimC and URI1_C_ subunits cooperate with other chaperones and/or co-chaperones including HSP90, HSP70 and HSP40, R2TP (Rvb1, Rvb2, Tah1, Pih1) and TRiC/CCT to help cells to cope with various stresses and in this manner to support the normal operation of a broad spectrum of cellular activities [11–14].

URI1 is referred to as an unconventional PFD since it is the only member of this family that possesses, besides all structural features of an α-class PFD, an about 200 amino acid long C-terminal extension [5]. This segment harbours specific binding sites for RPB5/POLR2E (polymerase RNA II DNA-directed polypeptide E) [15] and PP1γ (protein phosphatase 1, catalytic subunit, gamma isoform) [16] to mediate the assembly of RNA polymerases and to increase S6K1 survival signaling, respectively. Amplification and/or deregulated expression of URI1 has been observed in various cancer contexts including ovarian and hepatocellular carcinoma and multiple myeloma, supporting the view that URI1 may act as a multifaceted modifier of cancer cell proliferation and survival [17–22].

Given the structural and functional relationships of URI1 with molecular chaperones, we hypothesized that in this function, URI1 may help cancer cells to cope with the stress associated with oncogenic transformation. Accordingly, certain cancer cells may have evolved a specific dependency on the URI1 chaperone system for their survival. Here, we investigated whether such vulnerabilities exist in the context of colorectal cancer (CRC) cells.

## RESULTS

### Differential requirement of URI1 function for survival of CRC cell lines

To assess whether URI1 is amplified or not in human CRC cell lines, we analyzed copy number variation (CNV) data of *URI1* in the cancer cell line encyclopedia (CCLE, http://www.broadinstitute.org/ccle/home). This analysis revealed a lack of amplification of the *URI1* locus in CRC cell lines (Supplementary Table S1). This observation also extended to human CRC samples represented in The Cancer Genome Atlas of the TCGA Research Network (GISTIC results accessed through Tumorscape; http://www.broadinstitute.org/tumorscape) (Supplementary Table S2). A similar analysis performed for the gene encoding STAP1, the α-class PFD partner of URI1, revealed also no evidence of amplification in human CRC (data not shown).

**Figure 1:**
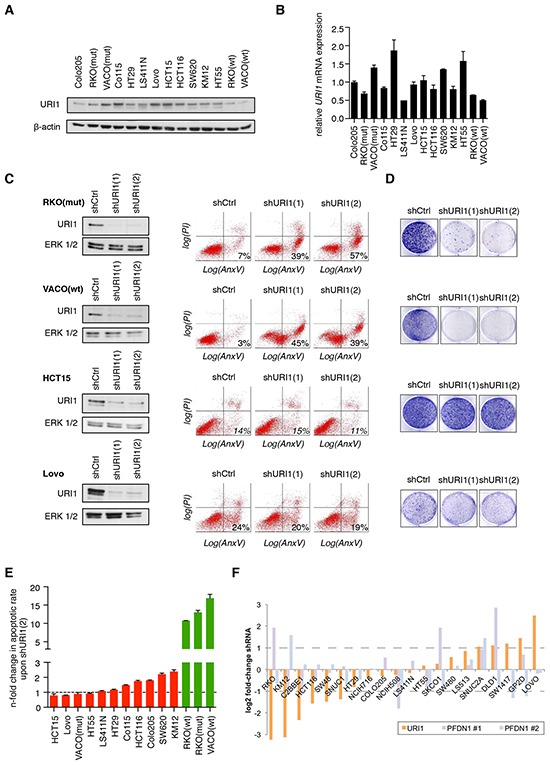
Differential requirement of URI1 function for the survival of CRC cell lines **A.** Representative immunoblot showing URI1 expression in a panel of 14 CRC cell lines (n=2). β-actin served as loading control. **B.** mRNA expression of URI1 across the same panel of CRC cell lines as in (A). by quantitative RT-PCR (qPCR). Data were normalized to TBP and GAPDH, and Colo205 URI1 mRNA levels were used as arbitrary reference sample (set to 1.0). Data are shown as means of three biological replicates, ± SD. **C.** Left panel: Immunoblots for URI1 in indicated CRC cell lines after targeting URI1 with two different shRNAs or a control shRNA. Total ERK1/2 served as loading control. Right panel: Representative apoptosis measurements of cells described in the left panel using Annexin V-GFP and propidium iodide (PI) double-staining (n=3). **D.** Representative clonogenic assays of cells described in (C; left panel) (n=3). **E.** Quantification of apoptotic responses in indicated CRC cells upon shURI1(2)-mediated URI1 depletion. Green and red denote URI1-dependent and -independent CRC cell lines, respectively. Error bars represent SD of three independent experiments taken from Supplementary Figure 1A. **F.** Achilles database: sensitivity of colorectal cancer cell lines to *URI1* and *PFDN1* shRNAs. *PFDN1* #1 and #2 denote the two significant shRNA sets identified by the ATARiS algorithm for *PFDN1*. Dotted lines represent a two-fold change.

In the absence of any indication of *URI1* amplification in human CRC, we assessed the effects of URI1 depletion on cell survival in a panel of 14 CRC cell lines with different mutational background and varying levels of URI1 protein and mRNA expression (Figure 1A and 1B). This cell line panel was infected independently with two specific shRNAs targeting URI1 [shURI1(1) and shURI1(2)] and the extent of apoptosis quantified by flow cytometry using combined Annexin V/propidium-iodide (PI) staining. Figure 1C illustrates the differential effects of URI1 depletion in four CRC cell lines. While RKO(mut) and VACO(wt) were highly dependent on URI1 function for their survival, HCT15 and Lovo were not. URI1 depletion also reduced colony formation of RKO(mut) and VACO(wt), but not of HCT15 and Lovo cells (Figure 1D). Analysis of the apoptotic responses to URI1 depletion in 10 additional CRC cell lines revealed that some cell lines display no or low, some moderate and yet other high dependency on URI1 for their survival (Supplementary Figure S1A). Thereafter, we refer to these distinct groups as URI1-independent (red bars) and URI1-dependent (green bars) CRC cell lines (Figure 1E). To corroborate that this differential vulnerability of certain CRC cells is not the result of insufficient downregulation of URI1 in URI1-independent cells, we combined shRNAs and siRNAs targeting URI1. The latter combination was highly efficient in depleting URI1 in the URI-independent cell line Co115 as evidenced by immunoblotting (Supplementary Figure S1C). Importantly, this treatment did not significantly increase cell death (Supplementary Figure S1B), indicating that URI1-dependency is not the result of an inefficient depletion but rather an intrinsic property of certain CRC cells.

The Achilles data portal catalogs results from genome-scale interrogation of gene function using pooled lentiviral shRNAs across hundreds of cancer cell lines (http://www.broadinstitute.org/achilles) [23]. A search for PFD genes and their impact on CRC cell lines revealed data only for URI1 and PFD1 in 20 CRC cells lines, of which 8 were also in our cell line panel. Intriguingly, across these 20 CRC cell lines, some cell lines demonstrated no or low, some moderate and yet other high dependency on URI1 for their survival (Figure 1F) thus corroborating our findings of the existence of a differential requirement for URI1 for CRC cell survival. The results of knockdowns of PFD1 by two distinct shRNA pools provided in the Achilles data portal show pattern of dependency for survival distinct to URI1. We note that ample biochemical evidence suggest that PFD1 is not part of the URI1 complex [4–7]. Data on other components of the URI1_C_ are currently not contained in the Achilles database. To extend this analysis, we asked next whether the URI1 survival dependency pattern identified correlates with genes that represent signaling network components to which URI1 has been previously functionally connected. This includes the PI3K-mTORC-S6K pathway [16] and the R2TP complex important for the assembly of RNA polymerases and PI3K-related protein kinases [5, 11, 12]. To this end, we established a list of representative genes corresponding to each of these networks (Supplementary Table S3). Among these selected genes, those contained in the Achilles database were used to compute the correlation in sensitivity with respect to URI1 across 20 CRC cell lines but, following adjustment for multiple hypothesis testing, none reached statistical significance (Supplementary Figure S1D). We note however a trend that the sensitivity of CRC cell lines to shRNAs targeting the PI3K-mTORC-S6K axis is inversely correlated to URI1 sensitivity (Supplementary Figure S1D). Finally, we explored whether known frequent genomic alterations in CRC correlate with URI1 sensitivity. By taking the 8 most frequently mutated genes in CRC (derived from The Cancer Genome Atlas Network [24]), we performed a computational analysis and found that, of these, *KRAS*, *TP53*, and *FBXW7* displayed the largest differences in mutation frequency between URI1-sensitive and URI1-insensitive cell lines (Supplementary Figure S1E). Neither of these differences were statistically significant after adjustments for multiple hypothesis testing (Fisher exact test with pFDR correction). Interestingly however, *KRAS* mutations, when restricted to the G12 or G13 positions, were found in 7 out 9 URI1-insensitive CRC cell lines but only in 2 out of 12 URI1-sensitive cell lines (pFDR value of 0.0644). This result may possibly imply that URI1 sensitivity is dependent on *KRAS* status in CRCs.

### URI1-dependent CRC cell lines are also dependent on the α-class PFD STAP1

**Figure 2:**
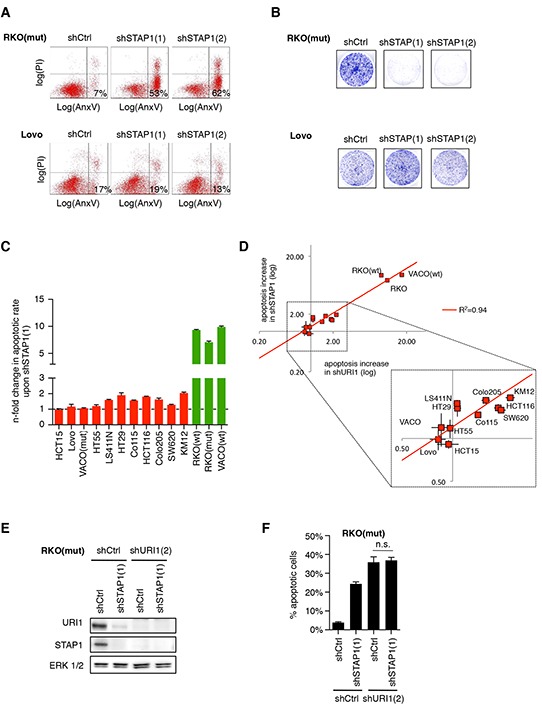
URI1-dependent CRC cells are also dependent on the α-class PFD STAP1 **A.** Representative apoptosis measurements of indicated CRC cells by Annexin V-GFP and propidium iodide double-staining after targeting STAP1 with two different shRNAs or control shRNA (n=3). **B.** Representative clonogenic assays of cells described in (A) (n=3). **C.** Quantification of apoptotic responses in indicated CRC cells upon shSTAP1(1)-mediated STAP1 depletion. Apoptosis increase relative to shCtrl following shRNA-mediated STAP1-depletion is displayed. Data are means of three biological replicates taken from Supplementary Figure S2B, ± SD. **D.** Relative levels of apoptosis induced by URI1 or STAP1 depletion in a panel of 14 CRC cell lines. Each cell line is represented by a pair of data points reflecting the two shRNAs used against each gene. **E.** Immunoblots for indicated proteins after expression of shRNAs targeting URI1, STAP1 or both. **F.** Apoptotic response measurements by Annexin V-GFP/PI co-staining in cells corresponding to E. Data are shown as means of three biological replicates, ± SD.

Next we explored whether URI1's requirement for cell survival of a subset of CRC cell lines relates to its role as a component of the ‘prefoldin-like’ URI1_C_. To assess this, we also depleted URI1's direct α-PFD binding partner STAP1 with two specific shRNAs [shSTAP1(1) and shSTAP1(2)] in CRC cells (Supplementary Figure S2A). As exemplified in Figure 2A, only the URI1-dependent cell line RKO(mut), but not the URI1-independent cell line Lovo, underwent cell death upon STAP1-depletion and also failed in colony formation (Figure 2B). Furthermore, across the panel of 14 CRC cell lines, cell death provoked by STAP1 depletion with two distinct shRNAs mirrored that of URI1 depletion (Figure 2C and 2D, Supplementary Figure S2B and S2C). At the biochemical level, depletion of STAP1 was paralled by loss of endogenous URI1 protein and vice versa (Figure 2E), suggesting that STAP1 and URI1 are reciprocally required for each other's protein expression. In line with this observation, the extent of cell death in RKO(mut) cells observed upon combined depletion of URI1 and STAP1 was similar to the extent of cell death observed upon depletion of URI1 alone (Figure 2F). That URI1 and STAP1 act as part of the URI1_C_ to mediate survival of a subset of CRC cell lines suggests that certain cancer cells have evolved a specific dependency on this class of molecular chaperone complexes.

### URI1 suppresses p53 activation in URI1-dependent CRC cells

Among frequent genetic alterations interfering with apoptosis in human cancers including CRCs are mutations in the p53 tumor suppressor [24]. p53 is activated in response to a broad range of extrinsic and cell intrinsic stress signals to halt cell cycle progression and induce cell death [25]. Hence, we tested whether p53 is differentially activated in URI1-dependent RKO(mut) and VACO(wt) and URI1-independent Co115 and Lovo cells. These four cell lines are known to express wild-type p53. The functionality of the p53 pathway in these cells was experimentally confirmed by treatment with the DNA damaging agent etoposide. As shown in Supplementary Figure S3A, etoposide induced p53 protein expression in p53 wildtype CRC cells and concommitantly, known p53 target genes including *PUMA*, *NOXA* and *CDKN1A* were activated. No such response was seen in two p53-defective cell lines HT29 and LS411N [26] (Supplementary Figure S3A) (http://www.broadinstitute.org/ccle/home).

**Figure 3:**
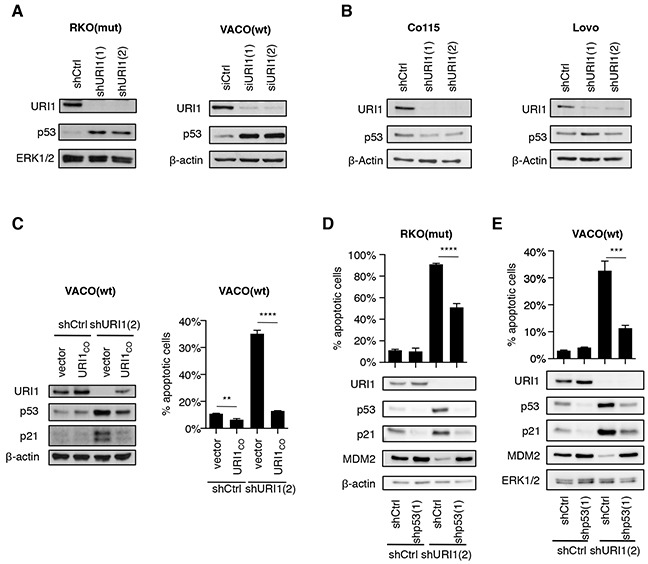
URI1 depletion causes activation of p53 in URI1-dependent CRC cells **A, B.** Immunoblots for indicated proteins upon URI1-depletion in URI1-dependent VACO(wt) and RKO(mut), and URI1-independent Co115 and Lovo cells, respectively. (n=3) **C.** Immunoblotting and measurement of apoptosis in VACO(wt) cells upon shURI1(2)-mediated URI1-depletion, with or without simultaneous re-expression of URI1 protein from a plasmid containing a URI1-codon optimized (URI1_CO_ ) coding sequence. (apoptosis assay n=3) **D, E.** Assessment of apoptosis levels and immunoblots for indicated proteins of URI1-depleted RKO(mut) and VACO(wt) cells, with or without concomitant p53-kd. Data are means of three biological replicates, ± SD.

Depletion of URI1 in the four p53 wildtype CRC cell lines mentioned above resulted in a profound accumulation of p53 in URI1-dependent but not -independent cell lines (Figure 3A and 3B). This effect and the associated apoptosis were both abolished by concurrent lentivirus-mediated re-expression of a URI1 cDNA harboring silent mutations (referred to as URI1_CO_) that enable escape from URI1-targeting shRNAs (Figure 3C). Moreover, URI1 depletion-induced p53 accumulation was the result of an increase in p53 protein stability as evidenced by cycloheximide-chase experiments (Supplementary Figure S3B). Importantly, in those CRC cells, in which URI1 depletion caused p53 activation, it also induced cell death in a p53-dependent manner as evidenced by the fact that the simultaneous downregulation of URI1 and p53 suppressed, to a significant extent, albeit not fully, cell death (Figure 3D and 3E). Similarly, we observed in these samples also induction of p21, downregulation of MDM2 and activation of p53 target genes, responses that were abolished by the simultaneous depletion of p53 (Figure 3D and 3E, Supplementary Figure S3C). Hence, p53 activation and p53-dependent cell death are specific consequences of impaired URI1 function in URI1-dependent CRC cell lines.

### Activation of p53 through disrupted URI1 function precedes DNA damage

**Figure 4:**
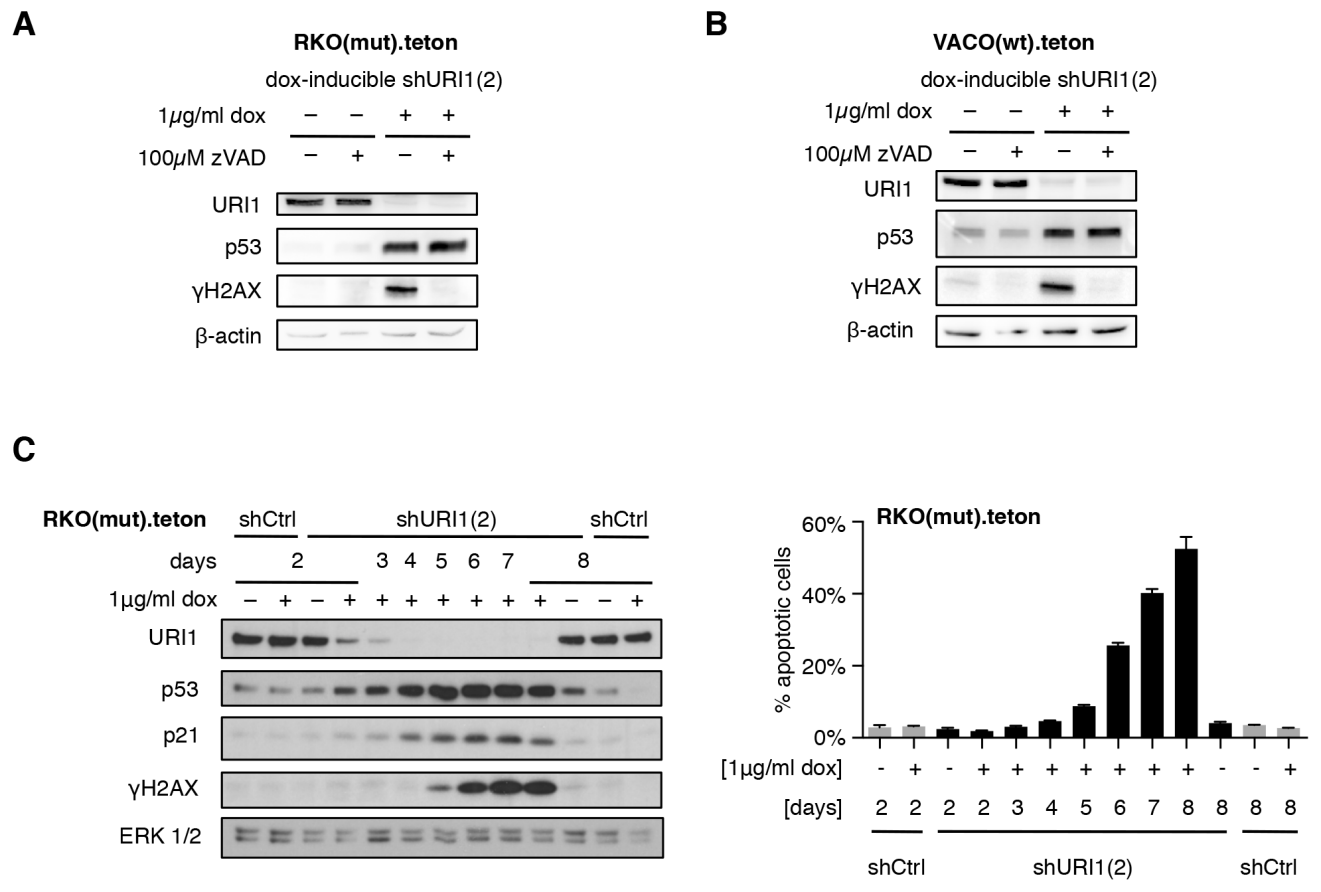
URI1 depletion-induced apoptosis causes γH2AX accumulation **A, B.** p53 activation and γH2AX accumulation upon URI1-depletion, in combination with blockade of apoptosis using 100μM of the pan-caspase inhibitor zVAD-FMK (or DMSO as vehicle control) in both RKO(mut) (A) and VACO(wt) (B) cells. (n=3) **C.** Immunoblots for URI1, p53, p21, γH2AX and ERK1/2 following URI1 depletion in RKO(mut) cells using a dox-inducible shURI1(2) construct (left), and the corresponding apoptosis levels (right). Data are shown as means of three biological replicates, ± SD.

To examine whether p53 activation is a consequence of URI1 depletion-induced DNA damage or not, we assessed phosphorylation of histone H2AX at S139 (referred to as γH2AX), a well-established read-out for DNA double-strand breaks [27], in RKO(mut) and VACO(wt) cells, in which URI1 was downregulated in a doxycycline-dependent manner. URI1 depletion caused strong induction of γH2AX (Figure 4A and 4B). This response was blocked by simultaneous treatment of these cells with the pan-caspase apoptosis inhibitor zVAD-FMK, but p53 protein remained induced (Figure 4A and 4B). As shown in Supplementary Figure S4, doxycyclin-induced downregulation of URI1 was accompanied by p53-dependent activation of the p53 target genes *NOXA*, *PUMA*, *CDKN1A* and *MDM2*. These observations indicate that the induction of γH2AX and apoptosis occur likely as a result of p53 activation and not through interference with URI1 function leading to a p53 stabilization secondary to apoptotic DNA damage.

To further corroborate this, we followed p53, p21 and γH2AX expression and corresponding extent of cell death in RKO(mut) cells over time. As shown in Figure 4C, as URI1 protein became depleted, p53 and p21 accumulated. Only at later time points, levels of γH2AX started to rise coinciding with the induction of cell death. These results demonstrate that in a subset of CRCs, URI1 loss promotes a cell death response that is mediated by and dependent on p53. URI1-independent CRC cell lines expressing wildtype p53 fail to execute such a response. In addition, the accumulation of γH2AX appears to be the result of DNA fragmentation that occurs during apoptosis and is therefore subsequent to URI1 loss-of-function-induced p53 activation in URI1-dependent CRC cells. Together, these data support a model, in which a root cause of differential p53-dependent apoptosis induction in CRC cells may be related to the disruption of URI1_C_-regulated client protein assembly/folding pathways.

### URI1-dependent CRCs require URI1 function for tumor growth *in vivo*

**Figure 5:**
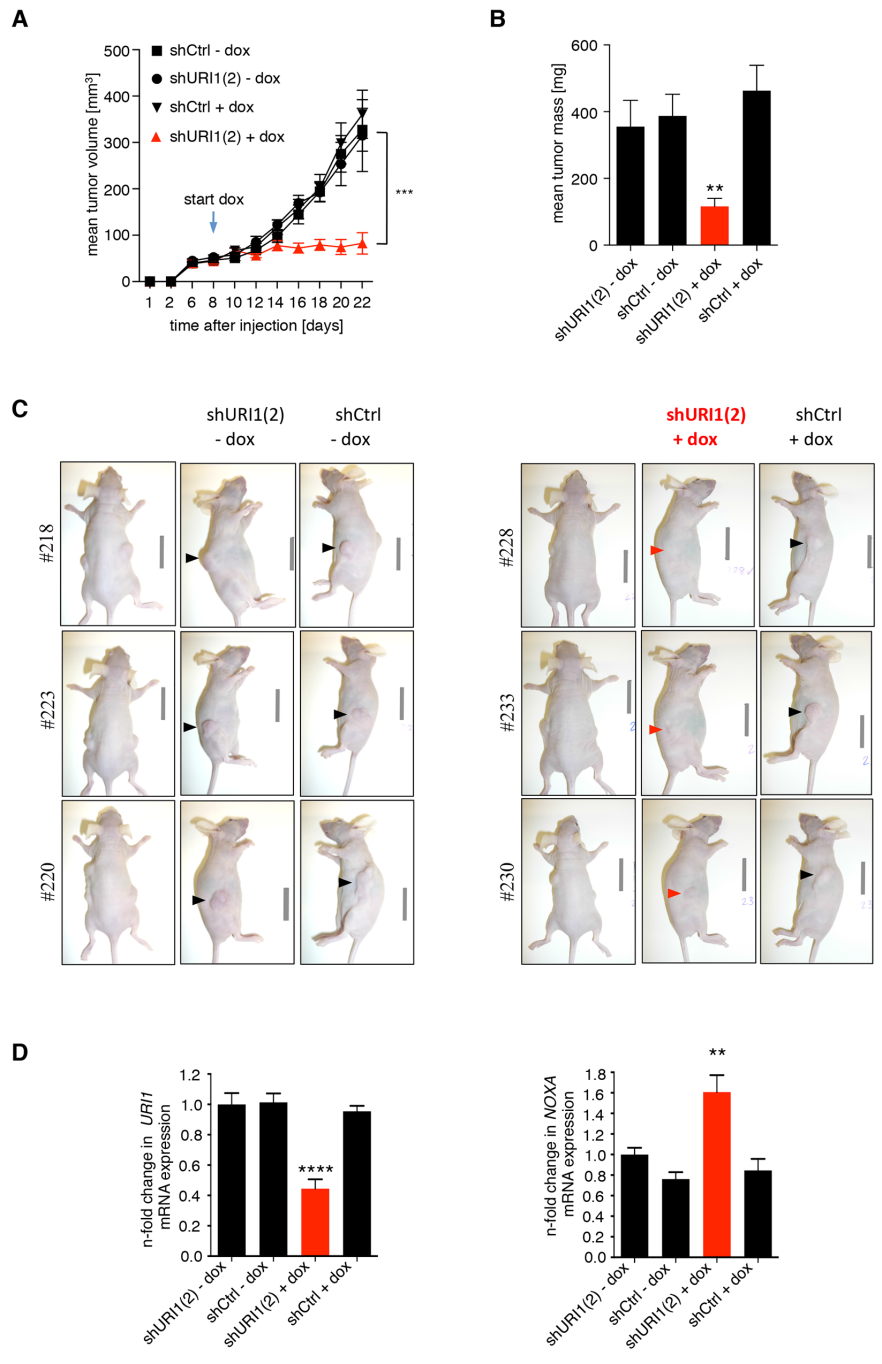
URI1 is required for tumor growth of URI1-dependent CRC cells after tumor establishment **A.** Mean tumor volume measurements of mice xenografted in both flanks with RKO(mut) cell line pools containing dox-inducible shURI1(2), as indicated. The arrow marks the start of feeding dox-containing chow to mice. n=8-10 flanks per group, error bars represent ± SEM, ***P ≤ 0.001. **B.** Mean tumor mass of explanted tumors, ± SEM, **P = 0.0025 **C.** A corresponding representative set of pictures of mice. **D.** qPCR analysis of tumors to assess URI1-kd efficiency and induction of the p53 target gene NOXA, n=4 tumors per group, mean values ± SD, ****P < 0.0001, **P = 0.0013. Values are normalized to geometric mean (geNorm) of TBP and 18S and shURI1(2) –dox (arbitrarily set as 1.0).

Given the importance of URI1 function for the survival of a subset of CRC cells *in vitro*, we asked whether this dependency also extends to an *in vivo* setting. To this end, we injected equal numbers of RKO(mut).teton.shURI1(2) or -shCtrl cells into the right and left flanks of immunocompromised Balb/c-nude mice, respectively. After successful engraftment when the tumor reached a certain size, one half of the mice received doxycycline (dox)-containing food. As illustrated in Figure 5A and 5B, RKO(mut).teton.shURI1(2) tumors displayed massively reduced tumor growth compared to shCtrl tumors or to tumors grown in mice on normal chow. Dox treatment did not affect the weight of mice over the time course of the experiment (Supplementary Figure S5). Explanted RKO(mut).teton.shURI1(2) tumors from dox-treated mice were on average much smaller than any of the control tumors (Figure 5B and 5C). Extraction of total RNA from four explanted tumors per group followed by qPCR analysis revealed reduced mRNA levels of *URI1* in URI1-depleted tumors compared to control tumors and increased expression of the p53 target gene *NOXA* in the corresponding samples (Figure 5D). Thus, URI1 function is required for maintenance of tumor growth and the suppression of the p53 target gene NOXA in URI1-dependent CRC cells *in vivo*.

## DISCUSSION

In this study we established the following experimental points. The unconventional PFD chaperone URI1 is selectively required for survival of a subset of CRC cell lines. Underlying this phenomenon is a property of URI1 to mediate cell survival, at least in part, through suppression of non-genotoxic activation of p53-dependent apoptosis both *in vitro* and in tumor xenografts. URI1-independent CRC cells do not activate p53-dependent apoptosis when URI1 function is compromised indicating that they have likely evolved a dependency on other cellular chaperone systems for their survival. The fact that STAP1-dependency of CRC cells parallels that of URI1 supports the view that this phenomenon is linked to URI1_C_ and thus represents a relevant chaperone function. These findings suggest that URI1 has properties of a ‘non-oncogene’ that supports the oncogenic phenotype of those cancer cells that have evolved a dependency on this molecular chaperone system for survival. Thus, depending on the cancer cell context, URI1 can mediate ‘oncogene addiction’ as is the case in carcinomas with *URI1* copy number increase [20] or ‘non-oncogene addiction’ as in CRCs, marking URI1 and the URI1_C_ attractive targets to be exploited for cancer therapy.

The very same CRC cell lines that rely on URI1 function for their survival, rely also on STAP1 for their viability. That depletion of URI1 caused concomitant loss of endogenous STAP1 and vice versa provides a biochemical explanation for the above-noted observation. It is also in line with the observation that the extent of apoptosis following depletion of either URI1 or STAP1 alone is similar to the levels of apoptosis seen after combined depletion of both α-PFD subunits. Deletion of single components from the classical prefoldin-/GimC-complex has been shown to cause degradation of the remaining subunits via a ubiquitin-proteasome pathway [28]. Thus, it is conceivable that the observed phenotypes associated with URI1 (and STAP1) depletion shown here are the result of impaired URI1_C_ function and consequent client protein misbehaviour.

The URI1_C_ engages with multiple client proteins to execute cell regulatory functions [3]. This provides a significant challenge for pinpointing precise mechanisms, through which silencing of URI1 leads to the activation of p53 and p53-dependent apoptosis in URI1-dependent CRC cell lines. However, URI1_C_ has been shown to associate with R2TP (Rvb1/2, Tah1, Pih1) and HSP90, which together form a larger complex important for the assembly and stability of phosphatidylinositol 3-kinase-related kinases (PIKKs), in particular mTOR and SMG1 [12, 29, 30]. Moreover, URI1_C_ has also been demonstrated to mediate the assembly of all three RNA polymerases, a function dependent on RPB5, a direct binding partner of URI1 and common subunit of RNA pol I, II and III [5, 11, 14, 31]]. Of note, there is ample evidence linking impaired function of either one of the three RNA polymerases to p53 activation and apoptosis [32–34]. Hence, the combined misbehaviour of these various URI1_C_ clients may, in the aggregate, contribute to the observed non-genotoxic p53 activation and p53-dependent apoptosis. Accordingly, in URI1-independent CRC cells, even in the setting of wildtype p53 expression, other chaperone systems may functionally substitute for the URI1_C_. Functional compensation through other chaperones has been observed in the case of HSP72 that can substitute for HSC70 in the context of HSP90 function [35].

Finally, emerging evidence suggests that tumor cells have evolved dependencies on specific molecular chaperone systems that protect them from proteotoxic stress and alongside provide specific vulnerabilities that can be exploited for anti-cancer therapy [1]. This is particularly well illustrated by work on members of the heat-shock protein (HSP) family. Increased expression of HSP90, for example, is a common feature of many cancers where it plays a key role in ensuring the proper folding, stability and activity of many client proteins. Drugs that block HSP90 ATPase activity such as 17-allykl-amino-17-demethoxygeldanamycin demonstrate potent anti-tumor activity due to, in part, drug-induced degradation of many cancer promoting HSP90-client proteins and the concurrent inhibition of multiple cancer-promoting signaling pathways [2]. The work presented here suggests that certain cancer cells have also evolved a dependency on a molecular chaperone system important for *de novo* protein folding for their survival. Thus, targeting specific interactions within the URI1_C_ may provide an attractive therapeutic opportunity.

## MATERIALS AND METHODS

### Cell lines and culture

The following colon cancer cell lines and culture media were used: RKO, VACO432 and HCT116 (B. Vogelstein, John's Hopkins University) and HT29 (Zurich Cancer Network's Cell Line Repository) were cultured in DMEM; LS411N, HCT15, Co115, Colo205 and KM12 (Zurich Cancer Network's Cell Line Repository) were cultured in RPMI; HT55 (ECACC) was cultured in DMEM-20; SW620 (Zurich Cancer Network's Cell Line Repository) was cultured in Leibo; Lovo (Zurich Cancer Network's Cell Line Repository) was cultured in F12. 293T embyronic kidney cells (ATCC) were cultured in DMEM. Cells from the Zurich Cancer Network's Cell Line Repository have undergone only a few passages since purchase from ATCC and are certified to be free from mycoplasma infection. DMEM = DMEM, high Glucose, NEAA (Gibco), supplemented with 2 mM L-Glutamine (Gibco) and 10% FCS (Ambimed). RPMI = RPMI 1640 + GlutaMax (Gibco), supplemented with 10% FCS (Ambimed). DMEM-20 = DMEM, high Glucose, NEAA (Gibco), supplemented with 2 mM L-Glutamine (Gibco) and 20% FCS (Ambimed). Leibo = Leibovitz's L-15 + GlutaMax (Gibco), supplemented with 10% FCS (Ambimed). F12 = DMEM/F12 + GlutaMax (Gibco), supplemented with 10% FCS (Ambimed). Cells were maintained at 37°C in a humidified atmosphere with 5% CO_2_.

### Colony formation assay

Cells were seeded in 6-well plates (Greiner) with 10'000 cells/well and cultured for 7 to 10 days until approximately 50 to 70% of the surface was covered in the wells with the highest confluence. Cells were then fixed and stained using crystal violet staining solution (0.5% w/v crystal violet, 70% methanol), washed using ddH_2_O and dried prior to scanning and quantification using the ImageJ software.

### Transfections

For transient transfection of siRNAs, Lipofectamine® RNAiMAX (Invitrogen) was used according to the manufacturer's instruction. URI1(1) (AACUUGUCCAUACUAAUGAAG) and URI1(2) (AAGGUAUCCUGAGUUACUUUG) siRNA sequences were as described previously [16]. sip53(1) and (2) sequences were AAGGAAAUUUGCGUGUGGAGU and UUGGUGAACCUUAGUACCUAA, respectively. The AllStars Negative Control siRNA from Qiagen was used as a non-targeting siRNA control. For plasmid transfections (to generate lentiviruses, see below), X-tremeGENE 9 DNA Transfection Reagent (Roche) was used according to the manufacturer's instruction.

### Generation and application of lentiviruses

For stable shRNA-mediated knockdown, pLKO-1 vectors from Sigma were purchased and lentiviral particles produced by co-transfection of the shRNA-containing vectors with 2^nd^ generation packing plasmids psPAX2 and pMD2G (Addgene clones 12260 and 12259, respectively) into 293T/17 cells. Virus was harvested after 48 hrs, filtered, and polybrene added to a final concentration of 8 μg/ml. Viral supernatants were used to infect target cells for 48 hrs and pools selected using 2 to 4 μg/ml puromycin, for 2 to 4 days. URI1 shRNA(1) and shRNA(2) were also subcloned into a blasticidin-containing pLKO-1 vector and pools selected using 10 to 20 μg/ml blasticidin for 4 to 6 days. For double knockdown, rescue or complementation experiments, double selection using both puromycin and blasticidin was employed. For URI1-kd, clones TRCN0000074239 = shURI1(1) and TRCN0000074242 = shURI1(2) were used. For p53-kd, TRCN0000010814 = shp53(1) and TRCN0000003755 = shp53(2), and for STAP1-kd, TRCN0000154852 = shSTAP1(1) and TRCN0000157240 = shSTAP1(2) were used. As control, pLKO-1 vectors (puromycin and blasticidin) containing a scrambled control shRNA were used. URI1 shRNA(2) was also sub-cloned into a doxycycline-inducible pLKO-1 vector [pLKO1.teton.shCtrl or -shURI1(2)], as described [36]. Doxycycline was purchased from Sigma Aldrich. For experiments using the pLKO1.teton system, tetracycline-free FCS was used and doxycycline-containing medium was replaced every 48 hrs.

For stable over-expression and kd-rescue experiments, a codon-optimized URI1 transcript (URI1_co_), which escapes RNAi, was cloned into a CMV-containing pLKO-1 vector (pLKO1-CMV::URI1_CO_). The construct differed in the shRNA-binding sequences as follows (spaces mark codons, silently mutated bases are shown underlined): shURI1(1)'s target sequence C TTG CCT GAT AAA TTG TCT T was mutated to A CTG CCC GAC AAA CTG TCA T, and shURI1(2)'s target sequence CT AAG AGG GTC CGA ATA AAT was mutated to CA AAG CGA GTC AGG ATT AAC in URI1_CO_.

### Annexin V and propidium iodide stainings

Cells harvested by trypsinization were washed with Annexin V binding buffer (10 mM Hepes/NaOH, pH 7.4, 140 mM NaCl and 2.5 mM CaCl_2_) and incubated with His-GFP-Annexin V-containing Annexin V binding buffer, as described previously [37]. Propidium Iodide (PI) was added just prior to assessing apoptosis by flow cytometry. Stained cells were analyzed using a BD FACSCalibur™ or BD Accuri™ C6 and the obtained data was analyzed with the FloJo software.

### Immunoblotting and antibodies

Immunoblotting was performed as described previously, on nitrocellulose membranes using a trans-blot turbo blotting device (Bio-Rad) [16]. Alternatively, 4-20% Mini-PROTEAN TGX Stain-Free Gels and Trans-Blot Turbo Transfer Packs (PVDF) were used, according to the manufacturer's protocols using a trans-blot turbo blotting device (Bio-Rad). The URI1 (mAb 58.1) and STAP1/UXT (mAb 105.128) antibodies have been described previously [5]. ERK1/2 (#9101) and P-Histone H2A.X S139 (abbreviated as γH2AX, #9718) antibodies were purchased from Cell Signaling Technology. MDM2 (sc-812) and p21 (sc-397-G) were purchased from Santa Cruz Biotechnology, β-actin (A5316) from Sigma Aldrich, and p53 (14211A) from BD Pharmingen.

### Chemicals

To inhibit apoptosis, the pan-caspase inhibitor zVAD-FMK (MBL International) was used. In long-term experiments, zVAD-FMK containing medium was exchanged every 48 hrs. The proteasomal inhibitor MG132 was purchased from Biomol. Cycloheximide (CHX) was obtained from Sigma-Aldrich.

### Quantitative real-time RT-PCR (qPCR)

Samples from cultured cells and explanted xenografts were processed using the RNA II kit (Macherey-Nagel) according to the manufacturer's protocol. 1 to 5 μg total RNA were transcribed using the RNA to cDNA EcoDry Premix (Random Hexamers) kit (Clontech). qPCRs were run on a Roche LightCycler LC480, using the following program: 5 min pre-incubation at 95°C, 40 amplification cycles (10 sec 95°C, 10 sec 60°C, 10 sec 72°C) and melting curve. Obtained data was analyzed using the Biogazelle qbase+ software. Employed PCR primers were: *HPRT1* (forward: CCTGGCGTCGTGATTAGTGAT; reverse: AGACGTTCAGTCCTGTCCATAA); *ACTB* (forward: CCTCGCCTTTGCCGATCCG; reverse: CCACC ATCACGCCCTGGTG); *GAPDH* (forward: GAAGGTG AAGTTCGGAGTC; reverse: GAAGATGGTGATGGG ATTTC); *RNA18S5* (forward: GTTCCGACCATAAAC GATGC; reverse: TGGTGGTGCCCTTCCGTCAAT); *TBP* (forward: TTCGGAGAGTTCTGGGATTGT; reverse: TGGACTGTTCTTCACTCTTGG C); HMBS (forward: ATGTCTGGTAACGGCAATGC; reverse: CGTCTGTAT GCGAGCAAGC); *URI1* (forward: AATGCCCTTCGA GAAAGACTCA; reverse: CCCCCAGTAAAACAGTG ACTTC); *BBC3/Puma* (forward: GACCTCAACGCAC AGTACGAG; reverse: AGGAGTCCCATGATGAGAT TGT); *PMAIP1/Noxa* (forward: GCAAGAACGCTCAA CCGAG; reverse: AAGTTTCTGCCGGAAGTTCA); *CDKN1A* (forward: AGTCAGTTCCTTGTGGAGCC; reverse: CATGGGTTCTGACGGACAT); *MDM2* (forward: TGTTGTGAAAGAAGCAGTAGCA; reverse: CCTGATCCAACCAATCACCT).

### Xenograft experiments

For xenograft experiments, six weeks old female BALB/cAnNRj-*Foxn1^nu/nu^* mice were purchased from Janvier. After two weeks of adaption to the mouse facility, 2 x10^6^ RKOmut.to.shCtrl or –shURI1(2) cells resuspended in PBS v/v 50% phenol-red free matrigel (BD Biosciences) were injected into the left and right flanks of 20 mice, respectively. After successful engraftment (8 days), mice were randomized to two groups and fed with chow containing 200 mg/kg doxycycline (doxycycline from Sigma-Aldrich; chow was produced and sterilized by Provimi-Kliba) and control chow, respectively. Mice were clinically observed and tumor sizes measured by caliper every two days. Tumor volumes were calculated as follows: vol = D * d^2^/2 with D being the greatest longitudinal diameter of the tumors, and d being the greatest transverse diameter [38]. At the end of the experiment, mice were sacrificed and the tumors explanted, weighed and snap frozen for extraction of total RNA (see section on qPCR).

Mean xenograft tumor volumes were analyzed by two-way RM ANOVA and Tukey correction for multiple testing, and means of body weights of mice were analyzed by two-way RM ANOVA. qPCR data of explanted xenograft tumors (n=4) were analyzed by ordinary one-way ANOVA and Dunnett's multiple comparisons test. Explanted tumor weights were analyzed by Kruskal-Wallis and Dunn's multiple comparisons test.

All animal work was performed in accordance with the guidelines of the institutional animal care and local veterinary office and ethics committee of the Kanton Zurich, Switzerland (reference number 149/2011) under approved protocols.

### Data presentation and analysis

All statistical analyses and data presentation were generated using the Prism 6 software (GraphPad Software Inc.) and R 3.2.3. Test details are given in the respective subsections (see below).

### Achilles analysis

Cell lines sensibility to shRNAs for URI1 and other genes as indicated were taken from the Achilles database v2.4 [23]. Values are ATARiS gene level scores (i.e. the average fold-change of shRNAs with consistent values across the 216 cell lines). Correlation between two genes was computed as the Pearson correlation. A pFDR correction for multiple hypothesis was applied when needed. URI1-sensitive cells were defined as cell lines with a log2 shRNA fold-change value below zero. Mutation status for the Achilles cell lines was taken from the Broad-Novartis Cancer Cell Line Encyclopedia (*http://www.broadinstitute.org/ccle*).

## SUPPLEMENTARY FIGURES AND TABLES


