# Supplementary material for: Colorectal cancer cells display chaperone dependency for the unconventional prefoldin URI1
Source: Oncotarget. 2016 Apr 18;7(20):29635–47. doi: 10.18632/oncotarget.8816 (PMC5045422; doi:10.18632/oncotarget.8816)
Supplement: Supplementary file 1 [file oncotarget-07-29635-s001.pdf]

SUPPLEMENTARY FIGURES AND TABLES

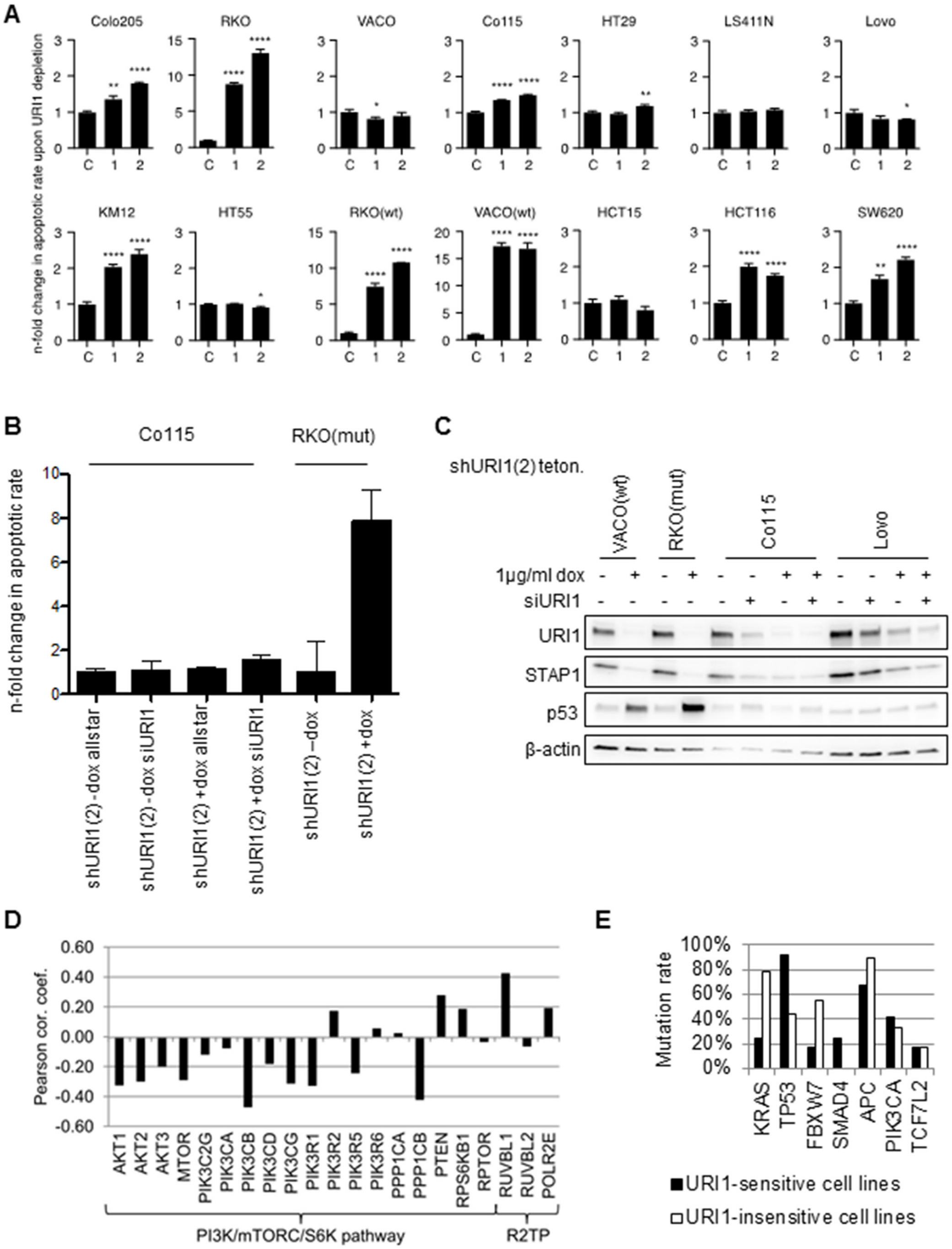

Supplementary Figure S1: Quabmntitative analyses of apoptotic responses of CRC cell lines to URI1 depletion. A. Quantification of the apoptotic response (assessed as in Figure 1C) in independent experiments across the entire panel of 14 CRC cell lines. C: shCtrl. 1: shURI1(1). 2: shURI1(2). Data are means of three biological replicates, ± SD. Values for shURI1(2) are grouped and shown in Figure 1E. **B.** Quantification of apoptotic response upon combined shRNA and siRNA targeting URI1 in the URI1-independent cell line Co115. Data are means of three biological replicates, ± SD. **C.** Immunoblots for indicated proteins upon combined URI1-depletion in URI1-independent Co115 and Lovo cells and shRNA only in the URI1-dependent cells VACO(wt) and RKO(mut), respectively (n=3). **D.** Correlation of URI1 sensitivity to shRNAs (Achilles database) and genes related to the PI3K/mTORC/S6K pathway and the R2TP complex. **E.** Mutation rate of the 8 most frequently mutated genes in CRC with respect to URI1-sensitivity in 21 CRC cell lines.

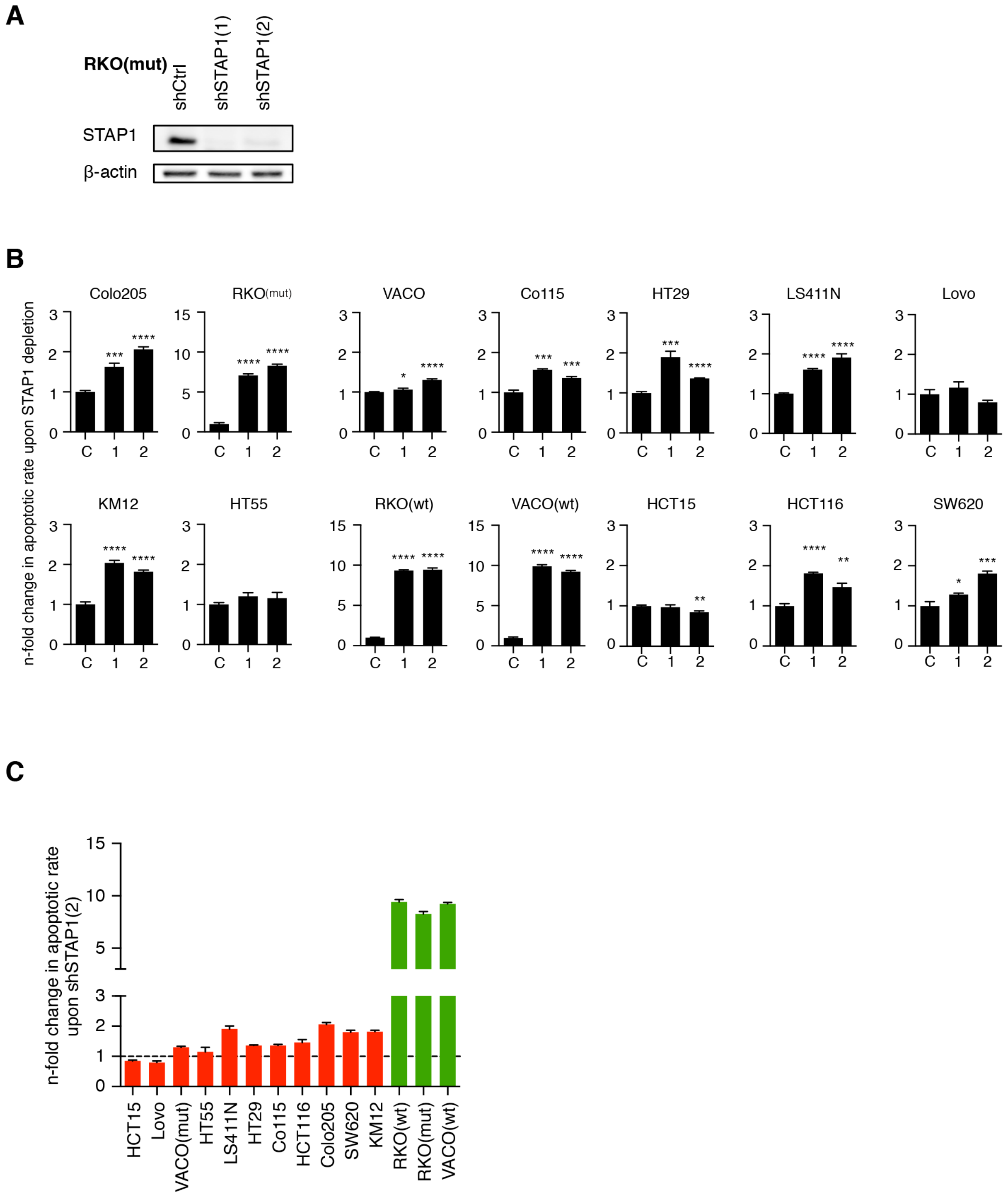

Supplementary Figure S2: Quantification of apoptotic responses of CRC cell lines to STAP1 depletion. A. Immunoblot for STAP1 after treatment with two different STAP1-targeting shRNAs in RKO(mut) cells. (n=3) **B.** Quantification of the apoptotic response upon STAP1-kd (assessed as in Figure 1C) in independent experiments across the entire panel of 14 CRC cell lines. C: shCtrl. 1: shSTAP1(1). 2: shSTAP1(2). Data are means of three biological replicates, ± SD. **C.** Values for shSTAP1(2) are grouped.

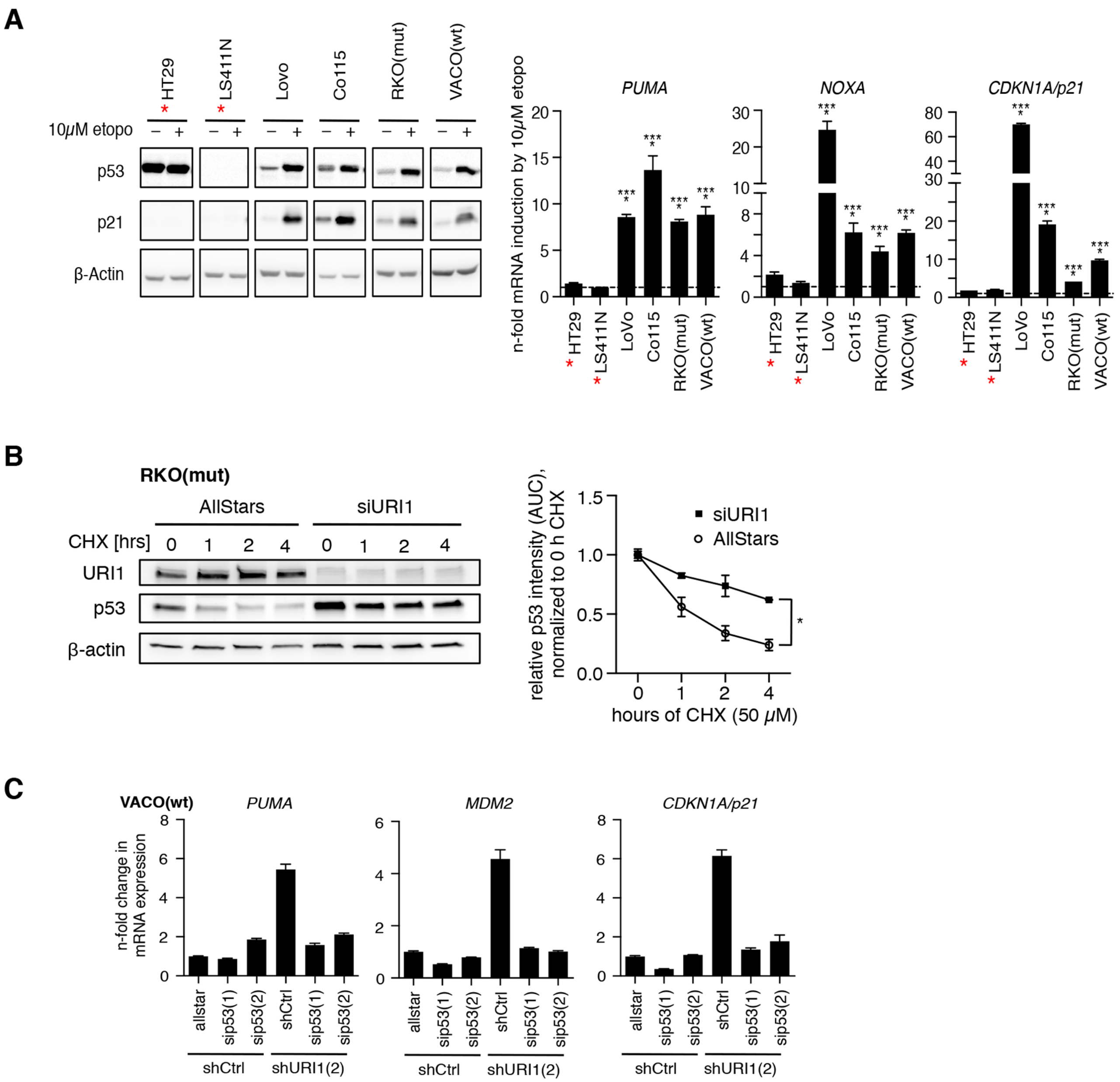

Supplementary Figure S3: Analyses of p53 responses as a function of URI1 depletion. A. Immunoblotting and qPCR analyses of p53 target genes of selected cell lines from the CRC cell line panel after treatment with etoposide (etopo) or vehicle control (DMSO) to assess functionality of the p53 response. Red star indicates cell lines HT29 and LS411N with mutant p53 and p53-null respectively. qPCR data are means of three biological replicates, ± SD. **B.** Assessment of p53 protein stability in RKO(mut) cells upon siRNA-mediated URI1-depletion versus scrambled siRNA control = AllStars. One representative immunoblot is shown (left), next to the quantification of two different immunoblots from two independent experiments to demonstrate changes in half-life (right). Data are means of those two quantifications, ± SD. **C.** Induction of mRNAs of selected p53 target genes upon URI1-depletion and concomitant p53 knockdown in VACO(wt). Data are shown as means of three replicates, ± SD.

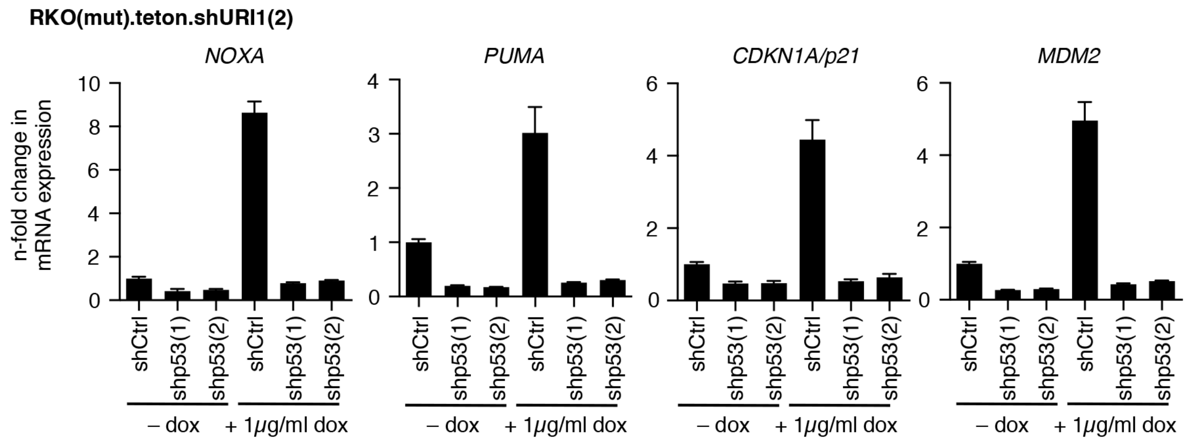

Supplementary Figure S4: Effects of URI1 depletion on p53 target genes. Induction of mRNAs of indicated p53 target genes upon doxycyclin-induced URI1-depletion and concomitant p53 knockdown in RKO(mut).teton.shURI1(2) cells. Data are shown as means of three replicates, ± SD.

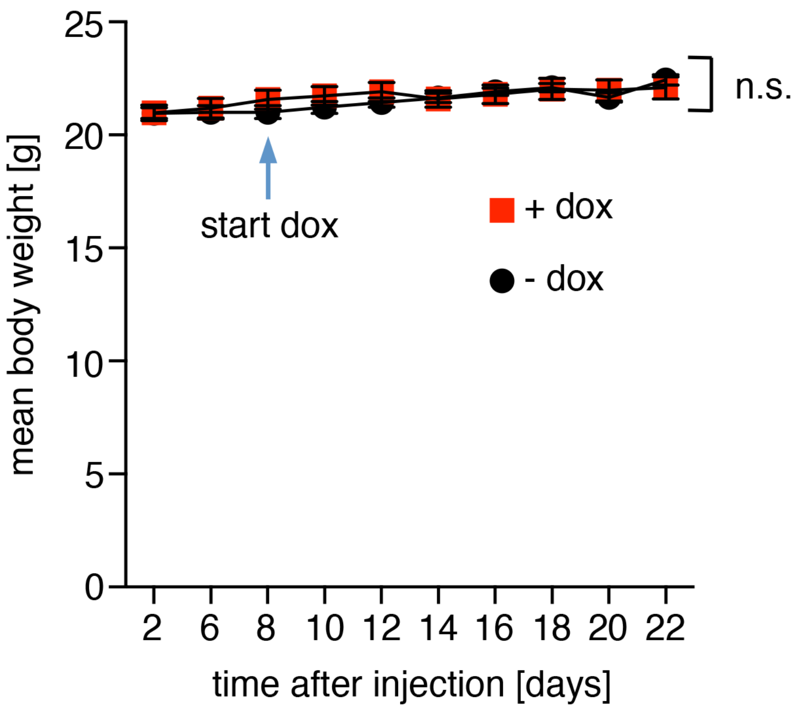

Supplementary Figure S5: Weight of tumor-bearing mice. Data are displayed as mean body weight of the two treatment groups, ± SEM.

Supplementary Table S1: Analysis of all CRC cell lines present in the CCLE database

cell line	URI1 CNV value
LS123_LARGE_INTESTINE	0.7137
SNU1033_LARGE_INTESTINE	0.6759
HT29_LARGE_INTESTINE	***0.3716***
HCC56_LARGE_INTESTINE	0.2498
SNU503_LARGE_INTESTINE	0.2367
HT55_LARGE_INTESTINE	***0.1670***
SW837_LARGE_INTESTINE	0.1496
LS1034_LARGE_INTESTINE	0.1426
OUMS23_LARGE_INTESTINE	0.1415
CL14_LARGE_INTESTINE	0.1278
SW948_LARGE_INTESTINE	0.1198
CCK81_LARGE_INTESTINE	0.1128
CL11_LARGE_INTESTINE	0.0465
C2BBE1_LARGE_INTESTINE	0.0437
NCIH747_LARGE_INTESTINE	0.0247
HS698T_LARGE_INTESTINE	0.0091
SNUC2A_LARGE_INTESTINE	0.0087
T84_LARGE_INTESTINE	0.0019
HS675T_LARGE_INTESTINE	0.0011
SNU1197_LARGE_INTESTINE	0.0002
KM12_LARGE_INTESTINE	***-0.0063***
MDST8_LARGE_INTESTINE	-0.0063
GP2D_LARGE_INTESTINE	-0.0094
SNU1040_LARGE_INTESTINE	-0.0124
SNU81_LARGE_INTESTINE	-0.0124
LS180_LARGE_INTESTINE	-0.0127
SNUC4_LARGE_INTESTINE	-0.0139
HRT18_LARGE_INTESTINE	-0.0143
CW2_LARGE_INTESTINE	-0.0262
HCT15_LARGE_INTESTINE	***-0.0266***
SNU175_LARGE_INTESTINE	-0.0286
SNU407_LARGE_INTESTINE	-0.0382
DLD1_LARGE_INTESTINE	-0.0405
CL40_LARGE_INTESTINE	-0.0417
SW1417_LARGE_INTESTINE	-0.0500

(*continued*)

cell line	URI1 CNV value
HCT116_LARGE_INTESTINE	***-0.0612***
SNUC1_LARGE_INTESTINE	-0.0679
SNUC5_LARGE_INTESTINE	-0.0713
SW48_LARGE_INTESTINE	-0.0736
RKO_LARGE_INTESTINE	***-0.0878***
HT115_LARGE_INTESTINE	-0.0924
SW1463_LARGE_INTESTINE	-0.1087
COLO205_LARGE_INTESTINE	***-0.1105***
LS513_LARGE_INTESTINE	-0.1120
LOVO_LARGE_INTESTINE	***-0.1136***
CL34_LARGE_INTESTINE	-0.1180
SW620_LARGE_INTESTINE	***-0.1372***
LS411N_LARGE_INTESTINE	***-0.1481***
SKCO1_LARGE_INTESTINE	-0.1880
SNU283_LARGE_INTESTINE	-0.1894
COLO678_LARGE_INTESTINE	-0.1996
NCIH508_LARGE_INTESTINE	-0.2128
SW480_LARGE_INTESTINE	-0.2817
COLO320_LARGE_INTESTINE	-0.3115
SW1116_LARGE_INTESTINE	-0.3659
NCIH716_LARGE_INTESTINE	-0.3703
SNU61_LARGE_INTESTINE	-0.4375
SW403_LARGE_INTESTINE	-0.4825
RCM1_LARGE_INTESTINE	-0.5033

Sorted by URI1 CNV value (descending order). Cell lines highlighted in bold and italics are present in our study.

Supplementary Table S2: TCGA analysis for *URI1*.

Cancer subset	In peak	Nearest peak	#Genes in peak	Q-value	Frequency of amplification		
					Overall	Focal	High-level
all_cancers	No	chr19:35002398-35098072	1	1.01E-27	0.1728	0.0562	0.0271
Ovarian	Yes	chr19:34455727-35347001	5	9.02E-4	0.2427	0.1068	0.0874
Breast	Yes	chr19:33310829-35346580	6	0.0203	0.2305	0.07	0.0329
Esophageal squamous	Yes	chr19:32764651-42274857	110	0.195	0.2727	0.2045	0.0455
all_epithelial	No	chr19:35002398-35098072	1	1.24E-28	0.2215	0.0694	0.0331
all_lung	No	chr19:35002398-35098072	1	1.05E-10	0.2584	0.084	0.0349
Lung NSC	No	chr19:35002398-35098072	1	1.89E-10	0.2469	0.0873	0.0355
Lung SC	No	No peak on chromosome	0	0.736	0.475	0.025	0.025
Prostate	No	No peak on chromosome	0	0.924	0.0652	0.0109	0.0109
Hepatocellular	No	No peak on chromosome	0	0.978	0.1983	0.0248	0.0
MPD	No	No peak on chromosome	0	1.0	0.0	0.0	0.0
all_neural	No	chr19:8920808-8939190	0	1.0	0.1475	0.0323	0.0
ALL	No	chr19:15641747-15670750	2	1.0	0.0077	0.0	0.0
Colorectal	***No***	***No peak on chromosome***	***0***	***1.0***	***0.1304***	***0.0062***	***0.0***
Glioma	No	No peak on chromosome	0	1.0	0.4878	0.122	0.0
Medulloblastoma	No	chr19:8920808-8939190	0	1.0	0.0859	0.0078	0.0
Melanoma	No	No peak on chromosome	0	1.0	0.1081	0.0090	0.0090
Renal	No	No peak on chromosome	0	1.0	0.0952	0.0159	0.0
all_hematologic	No	chr19:15651069-15670750	1	1.0	0.0229	0.0043	0.0029

CRC is highlighted in italics and bold. MPD = Myeloproliferative disorder. ALL = Acute lymphoblastic leukemia. ‘C19ORF2’ was used as term to query the TCGA database

Supplementary Table S3: Selected genes related to PI3K/mTORC/S6K pathway and the R2TP complex

Gene symbol	Pathway / complex	In achilles
AKT1	PI3K-mTORC-S6K	yes
AKT2	PI3K-mTORC-S6K	yes
AKT3	PI3K-mTORC-S6K	yes
MLST8	PI3K-mTORC-S6K	no
MTOR	PI3K-mTORC-S6K	yes
PIK3C2A	PI3K-mTORC-S6K	no
PIK3C2B	PI3K-mTORC-S6K	no
PIK3C2G	PI3K-mTORC-S6K	yes
PIK3C3	PI3K-mTORC-S6K	no
PIK3CA	PI3K-mTORC-S6K	yes
PIK3CB	PI3K-mTORC-S6K	yes
PIK3CD	PI3K-mTORC-S6K	yes
PIK3CG	PI3K-mTORC-S6K	yes
PIK3R1	PI3K-mTORC-S6K	yes
PIK3R2	PI3K-mTORC-S6K	yes
PIK3R3	PI3K-mTORC-S6K	no
PIK3R4	PI3K-mTORC-S6K	no
PIK3R5	PI3K-mTORC-S6K	yes
PIK3R6	PI3K-mTORC-S6K	yes
PPP1CA	PI3K-mTORC-S6K	yes
PPP1CB	PI3K-mTORC-S6K	yes
PPP1CC	PI3K-mTORC-S6K	no
PTEN	PI3K-mTORC-S6K	yes
RPS6KB1	PI3K-mTORC-S6K	yes
RPS6KB2	PI3K-mTORC-S6K	no
RPTOR	PI3K-mTORC-S6K	yes
PDRG1	R2TP	no
PFDN2	R2TP	no
PFDN6	R2TP	no
PIH1D1	R2TP	no
POLR2E	R2TP	yes
RPAP3	R2TP	no
RUVBL1	R2TP	yes
RUVBL2	R2TP	yes
UXT	R2TP	no
WDR92	R2TP	no
